# Predicting Problem Gambling in Young Men: The Impact of Sports Gambling Frequency and Internalizing Symptoms

**DOI:** 10.1007/s10899-025-10403-0

**Published:** 2025-06-11

**Authors:** Vincent O. Mancini, Jack D. Brett, Robert M. Heirene, Krista Fisher, Thom P. Nevill, Francis Mitrou

**Affiliations:** 1 Human Development and Community Wellbeing, The Kids Research Institute Australia, Perth, WA Australia; 2 Centre for Child Health Research, The University of Western Australia, Perth, WA Australia; 3 Brain & Mind Centre, School of Psychology, Science Faculty, University of Sydney, Sydney, NSW Australia; 4 Orygen, Parkville, VIC Australia; 5 Centre for Youth Mental Health, The University of Melbourne, Melbourne, VIC Australia; 6 Movember Institute of Men’s Health, Melbourne, Australia

**Keywords:** Gambling, Young men, Mental health, Sports gambling, Male health

## Abstract

Young men aged 18–25 years are at disproportionately increased risk for gambling problems compared to their older or female counterparts. The unique mechanisms that precipitate these problems in this group remain unclear. Data from the largest longitudinal cohort study on Australian men’s health (the *Ten to Men* Study) were used to identify the psychosocial, health-related, and gambling-related behavioral predictors of problem gambling severity in 265 young men aged 18–25 years. Hierarchical multiple ordinal logistic regression analyses found these predictors to explain a moderate proportion of variance in problem gambling severity. Four of the 17 predictors included accounted for unique variance. Specifically, higher levels of problem gambling severity were explained by lower levels of anxiety, higher levels of depression, and a higher frequency of prior engagement in *sports* gambling and *fantasy sports* gambling. Other psychosocial factors (e.g., masculine norm adherence), health-related behaviors (e.g., problem alcohol use), and gambling-related behaviors (e.g., casino or electronic gaming machines) did not explain any further variance. These findings are the first to longitudinally examine the risk factors for problem gambling severity in Australian men aged 18–25 years. The findings highlight unique developmental and sociocultural factors that may increase young men’s vulnerability to problem gambling. Whilst mental health difficulties are a known risk factor for gambling pathology, the identification of sports and fantasy sports gambling frequency as the greatest risks for later gambling problems emphasizes a need for initiatives that can identify and support young men susceptible to this form of gambling.

**Supplementary Information:**

The online version contains supplementary material available at 10.1007/s10899-025-10403-0.

Participation in legal forms of gambling is a recreational pastime for many Australians. Recent estimates indicate that more than 73% of Australians gamble at least once a year, whilst 38% of the population gamble weekly (Australian Institute of Health & Welfare, 2023). This high frequency of gambling activity is also paired with significant economic losses. Australia reports the largest per capita gambling losses out of any nation, totaling more than $25 billion annually (Australian Institute of Health & Welfare, 2023). These financial losses are not evenly distributed amongst the population. Instead, past research has revealed that specific subgroups are at an increased risk for gambling-related harms. One group predisposed to increased risk are males aged 18 – 25 years (Nyemcsok et al., 2022). In this paper we define problem gambling risk from the severity categories under the Problem Gambling Severity Index (PGSI; Ferris, 2001), with “Problematic Gambling” referring to those individuals most likely to meet a clinical diagnosis threshold for gambling behavior per the Diagnostic and Statistical Manual of Mental Disorders Fifth Edition (DSM-5).

Gambling-related harms are not only quantified by the dollar amount lost but extend to psychological and social consequences, which researchers and policymakers have identified as an emerging and significant public health issue in Australia — particularly amongst young men.

## Young Men’s Gambling Behaviors

The frequency and pattern of gambling behavior is gendered. Australian data has revealed that ~ 80% of men have gambled within the past 12 months, compared to ~ 66% of women. Additionally, Australian men also participate in all forms of gambling more than women and simultaneously make larger wagers (Australian Gambling Research Centre, 2023). Research also suggests that young men are more likely to report engaging in multiple types of gambling, compared to young women (Latvala et al., 2019; McCormack et al., 2014). Men tend to demonstrate a stronger preference for gambling activities that feature a skill-based component (e.g., sports betting, card games such as blackjack) rather than activities that are highly-chance-based (e.g., the lottery) compared to their female peers (Baggio et al., 2018; Stevens & Young, 2010). To exemplify, recent data from the United States revealed that 72% of sports bettors were male (Statista, 2024). Relatedly, younger adults also show stronger preferences for skill-based forms of gambling compared to older adults. Thus, younger men may be particularly drawn toward gambling activities that feature a skill-based component (Stevens & Young, 2010). Research also indicates that young men are generally more likely to participate in internet gambling (e.g., online sports betting, online poker) compared to young women, whilst younger adults are more likely to gamble online than older adults (Hollén et al., 2020; Latvala et al., 2019). In a survey of the gambling behaviors of undergraduate university students, McBride & Derevensky (2012) report significantly higher rates of internet gambling among males (11.8%) than among females (0.6%).

Men are also at disproportionately increased risk for gambling-related harms, even after accounting for the increased frequency at which they gamble compared to women. For example, 53% of male gamblers, compared to 38% of female gamblers, were classified as ‘at-risk’ for gambling-related harm in a recent Australian report (Australian Institute of Health & Welfare, 2023). This finding is also congruent with international prevalence estimates which suggest this is stable across several nations (Carneiro et al., 2020; Tran et al., 2024). Some types of gambling are more closely related to problem gambling than others. For example, recent studies indicate that slot machine gambling and online gambling (e.g., online sports betting) are more likely to be associated with problem gambling than other types of gambling (Browne et al., 2023). In general, the features of products thought to be most likely to result in harmful patterns of gambling include fast speed of play, including the ability to make repeated bets or plays in quick succession; short event duration; the ability to stake varying amounts, including large sums of money; ease of access; and continuous availability (Parke et al., 2016). Online gambling is characterized by many of these features, including convenience and increased accessibility, as well as being more discreet. These features have been linked to increased risk and found to be viewed as attractive among young people (Gainsbury et al., 2013; Hing et al., 2024a; Hing, Thorne, et al., 2024a, b). Relatedly, the proliferation of online gambling marketing and promotion campaigns – particularly related to organized sports – through traditional media outlets (e.g., television) and social media has contributed to a current landscape which makes online gambling content difficult to avoid or opt-out of for many Australians.

Sociocultural determinants are thought to play an important role in explaining higher rates of gambling participation and problems in men. Gambling is characterized by the presence of risk, and men are more likely to seek out and participate in risk-taking behaviors (Carneiro et al., 2020; Merkouris et al., 2016). These are reinforced by the traditional Western construction of masculinity, where a proclivity for risk-taking is understood to be a key facet (Mahalik et al., 2003). Risk-taking behaviors also often peak in late adolescence and early adulthood (Josef et al., 2016). This aligns with research on gambling frequency across developmental stages that indicate that rates of gambling participation are highest in young adults (Hollén et al., 2020). Accordingly, there is compelling evidence that points toward males in early adulthood as especially vulnerable to the risk of gambling-related harms (Welte et al., 2011, 2015).

Australian research indicates that gambling companies regularly seek to leverage young men’s predisposition toward risky behaviors through promotional and marketing strategies appealing to this group (Gainsbury et al., 2015). Gendered marketing campaigns often feature younger men as the protagonists where gambling is portrayed as an opportunity to achieve higher social status, appear more desirable to the opposite sex, and build mateship (Guillou-Landreat et al., 2021; Nyemcsok et al., 2022). These strategies have contributed to the perception that young men’s gambling, especially on past times that are traditionally masculinized (e.g., organized sports), may not just be an accepted behavior, but an *expected* one (Lamont & Hing, 2020). This may serve to accelerate and maintain this group’s elevated risk for gambling-related harms.

### Determinants of Young Men’s Problem Gambling Severity

Identifying gender and age as risk factors for problem gambling provides little recourse for intervention given their immutability. Accordingly, there is a need to identify malleable factors that may protect young men from gambling problems. Healthcare providers and policymakers may be especially interested in these mechanisms to improve identification and intervention to help improve the prevention and intervention of gambling-related harm. This is particularly pertinent to young men, where additional barriers exist to seeking professional support (Seidler et al., 2024a, b), such as stigma surrounding men’s mental health, prior dissatisfaction with professional support deterring future intention, and the potential unmet need for masculinities-informed mental health services (Seidler et al., 2017, 2018a, b). Despite some research investigating the role of gender in Australian gambling behaviors (Hing et al., 2016), few have concentrated on the experiences of young men specifically. Even fewer have accomplished this using longitudinal data.

One recently published study by Mancini (2025) enlisted the use of longitudinal data to identify the psychosocial determinants of Australian men’s problem gambling severity. The sample constituted 2,018 Australian men with an average age of 44 years. Most of this sample (~ 90%) were older than 26 years of age. These results revealed that, after accounting for a range of demographic (age and socioeconomic status) and psychosocial factors (masculine norm adherence, wellbeing, anxiety, depression, problem alcohol use, and loneliness), only perceived social support and prior 12-month gambling frequency could explain unique variance in problem gambling severity. Specifically, poorer perceptions of social support and higher frequency of recent gambling behavior were associated with greater problem gambling severity. These results helped to fill an important gap in the availability of longitudinal and Australian data on men’s gambling behaviors. However, the relevance of these findings to the experiences of young men specifically, are not yet clear – despite gambling being identified as an issue that disproportionately impacts young men (Benakovic et al., 2024).

There are several reasons why a different pattern of findings may be anticipated among young Australian men (i.e., those aged 18–25 years) and why concentrated effort to examine the experiences of young men warrants interrogation. First, early adulthood presents social and developmental changes. For many, this period is characterized by the end of formal schooling, a shifting prioritization of peer relationships over parents and an introduction to previously prohibited behaviors, like the consumption of tobacco and alcohol and participation in gambling (Schulenberg et al., 1997). Second, the novelty of freely engaging in these risky behaviors may be especially appealing, as early adulthood is the stage in a person’s life when risk-taking behaviors will reach their peak (Steinberg et al., 2018). This period is also when men most strongly endorse traditional masculine norms (e.g., self-reliance, emotional restraint) that may be harmful to psychosocial wellbeing (Herreen et al., 2021; Rice et al., 2011). Third, this period also coincides with entry into the paid workforce and disposable income that may precede the development of financial literacy and responsibility. Last, social and generational shifts in Australian society mean that the experiences of young men living in the 2020s may have distinct differences from those from prior decades. Changes to gambling policy and legislation combined with emerging technologies have transformed how young men can gamble. To exemplify, online gambling has only been legal in Australia since 2001. Moreover, the advent of the smartphone (~ 2007) gave rise to the development of instant access betting applications previously inaccessible to earlier cohorts of young men, whose gambling behaviors were typically restricted to physical attendance at select venues, or by telephone call to a bookmaker or betting agency. Past evidence suggesting that young adults gamble differently to older adults might also mean that the factors that influence their gambling habits (including risk for gambling problems) are also different. For example, younger adults more often engage in interactive and skill-based forms of gambling compared to older adults who prefer traditional chance-based forms such as electronic gambling machines (Garzola, 2024). Accordingly, if the mechanisms that contribute to young men’s problem gambling behaviors and the platforms through which they are more likely to gamble (i.e., online vs in venues) are unique from other cohorts, then treating these problems effectively may require tailoring.

### The Present Study

This study aimed to provide an account of the psychosocial, health-related, and behavioral predictors of problem gambling severity in young Australian men aged 18–25 years, using data from the nation’s largest cohort study on Australian male health and wellbeing. Enlisting similar methods to those recently reported by Mancini (2025), we hypothesized that:

- **H1:** Variables spanning five broad domains of functioning (i.e., demographics, masculine norm conformity, health-related behaviors, social connectedness and mental health, and prior gambling frequency) will predict a significant proportion of variability in young men’s problem gambling severity.
- **H2:** These predictors will each uniquely account for significant variance in problem gambling severity.

## Methods

### Participants

The participants consisted of 265 young men from the Ten to Men (TTM) Study (Currier et al., 2016). A power analysis was conducted using G*Power (Faul et al., 2009), indicating that the required sample size to achieve 80% power to detect a small to medium effect (i.e., ΔR^2^ = 0.06), was *N* = 244 for our hierarchical multiple regressions (e.g., 18 total predictors with 8 of interest [gambling type]). TTM is a cohort study funded by the Australian Commonwealth as part of an initiative to improve the health of Australian males and make the health system more responsive to their needs (Pirkis et al., 2017). The TTM Study has collected data from men across four waves: Wave 1 (Oct 2013 – Jul 2014), Wave 2 (Nov 2015 – May 2016), Wave 3 (Jul 2020 – Feb 2021), and Wave 4 (Aug 2022 – Dec 2022). To be eligible for the current study, men had to be 25 years of age or younger at Wave 3, complete the current outcome variable (i.e., problem gambling severity) at Wave 4, and complete predictor variables at either Wave 1, 3, or 4 (see supplementary materials for a visual depiction of sample selection). Demographic descriptors of the young men sample can be found in Tables 1 and 2.

**Table 1:** Demographic variables for our young men sample (*n* = 265)

Variable	% Yes
Highest Level of Education Achieved	
Still attending high school	2.3%
Did not complete Year 12	3.5%
Completed Year 12	42.4%
Cert I/II or other non-school qualification	6.1%
Cert III/IV (including trade certificate)	16.4%
Advanced diploma / diploma	6.9%
Bachelor’s degree (with or without honors)	21.0%
Graduate diploma / graduate certification	0.4%
Postgraduate degree	1.1%
Paid employment	84.7%
Living situation	
Living alone	4.9%
Living with parent(s)	58.9%
Living with other adult(s)	24.7%
Living with sibling(s)	33.1%
Living with partner	19.8%
Identified as Aboriginal and/or Torres Strait Islander	1.9%
States/Territories	
New South Wales	31.3%
Victoria	32.4%
Queensland	17.4%
South Australia	3.9%
Western Australia	10.4%
Tasmania	1.9%
Northern Territory	0.8%
Australian Capital Territory	1.9%

**Table 2:** Descriptive statistics of the interest variables for Australian men aged 18–25 years (*n* = 265) and Australian men aged above 25 years (*n* = 1,838)

	*M* (*SD*) of current sample	Skewness	Kurtosis	Cronbach’s α	M (SD) of older men
PGSI (Wave 4)	1.00 (2.63)	4.63	28.9	.89	0.68 (1.94)
Age (Wave 3)	21.62 (2.25)	0.04	−1.24	-	-
SES (Wave 4)	6.28 (2.8)	−0.28	−1.12	-	5.95 (2.71)
CMNI	**29.26 (5.48)**	**0.07**	**−0.10**	**-**	27.6 (5.24)***
Wave 1 (*n* = 160)	29.29 (5.42)	0.03	0.09	.63	-
Wave 3 (*n* = 107)	29.21 (5.58)	0.14	−0.43	.72	-
AUDIT (Wave 3)	8.35 (4.81)	0.75	0.21	.77	8.03 (5.63)
Loneliness (Wave 3)	**10.38 (3.96)**	**0.17**	**−0.72**	**.89**	8.85 (3.68)***
Social support (Wave3)	**30.52 (8.04)**	**−0.83**	**0.05**	**.97**	29.4 (8.63)*
Wellbeing (Wave 3)	71.75 (16.47)	−1.01	0.80	.89	72.2 (15.0)
Anxiety (Wave 3)	**4.79 (4.85)**	**1.21**	**0.74**	**.91**	3.25 (3.89)***
Depression (Wave 3)	**6.36 (5.76)**	**1.21**	**0.91**	**.90**	4.12 (4.44)***
	% Yes				% Yes
Smoking—Cigarettes (Wave 3)	16.2%	-	-	-	14.9%

*PGSI* Problem Gambling Severity Index, *SES* Socioeconomic status, *CMNI* Conformity to masculine norms inventory, *AUDIT* Alcohol Use Disorders Identification Test score; See section Materials for how all variables were measured. Chi-square significance testing was calculated to investigate whether predictor variables differed in our sample of young men aged 18–25 years compared to their older counterparts (25 years +). Bold values indicate significant differences. *p < .05; **p < .01; ***p < .001

### Materials

#### Problem Gambling Severity.Gambling Severity

The 9-item problem gambling severity index (PGSI) by Ferris (2001) was used to assess participant problem gambling symptoms. This measure was administered to participants at Waves 3 and 4 of the TTM Study. Participants responded to each item using a four-point Likert scale ranging from (0) *never* to (3) *always*. Given this variable's high skewness and kurtosis (see Table 2), the categories suggested by Holtgraves (2009) were used to identify the risk of problematic gambling severity. These categories were: *No Risk (score of 0)*; *Low Risk (1–2)*, *Moderate Risk (3–7)*, and *Problematic Gambling (8* +*)*. The PGSI showed good internal reliability in the current sample (see Table 2).

#### Age

Participant age (in years) at Wave 3 was derived from the reported date of birth, confirmed at each wave of the study.

#### Socioeconomic Status (SES)

Participant SES was measured using the Socio-Economic Indexes for Areas (SEIFA) as recorded by the Australian Bureau of Statistics (Australian Bureau of Statistics, 2018). Higher decile scores (derived from participant postal code) indicate areas of greater socioeconomic advantage and serve as a proxy for individual-level SES. This measure was administered to participants at all Waves of TTM; our study uses Wave 3 report.

#### Conformity to Masculinity Norms

Men’s conformity to traditional Western conceptualization of masculinity was assessed using the conformity to masculine norms inventory-22 (CMNI-22) by Owen (2010). This 22-item measure spans 11 dimensions of masculine norms (e.g., risk-taking, self-reliance, pursuit of status) that are routinely summed to produce an overall score. Participants respond to each item on a four-point Likert scale ranging from (0) *strongly disagree* to 3 *strongly agree*. Higher scores are indicative of greater conformity to these masculine norms. The measure was administered to adult participants at Wave 1. Participants who were under 18 years of age at Wave 1 then completed the measure at Wave 3 (except for two participants who completed it at both time points; their Wave 3 value was used in analyses). The overall score on the CMNI-22 showed slightly below adequate and adequate internal reliability in Waves 1 and 3, respectively (Table 2). This instrument has been previously used in published work studying the health of Australian men (Herreen et al., 2021).

#### Problem Alcohol Use

Total scores obtained from the World Health Organization’s Alcohol Use Disorders Identification Test (AUDIT) by Bush et al. (1998) were used to assess participants alcohol use at Wave 3. Participants respond to items on a 5-point Likert scale ranging from 0 *Never* to (4) *Daily or almost daily*. Higher scores indicate greater frequency of alcohol consumption. The AUDIT provided good internal reliability in the current study (Table 2). This measure was administered to participants at all Waves of TTM; our study uses Wave 3 report.

#### Smoking – Cigarettes

Participants reported whether they currently smoked cigarettes, measured as a binary outcome (Yes/No). This measure was administered to participants at all Waves of TTM; our study uses the Wave 3 report.

#### Loneliness

The four-item version of the UCLA Loneliness Scale (Russell et al., 1980) was used to assess loneliness symptoms. Participants responded to each item on a 5-point Likert scale from (1) *Never* to (5) *Always*. Higher scores indicate greater loneliness. The measure showed good internal reliability in the current study (Table 2). This measure was administered to participants at Wave 3 and Wave 4 of the TTM Study.

#### Emotional and Social Support

The 8-items of the Medical Outcomes Study (MOS) social support survey’s emotional/information subscale (Moser et al., 2012) were used to measure participant’s level of perceived social support, included in all Waves of the TTM Study. Participants respond to items on a 5-point scale ranging from (1) *none of the time* to (5) *all the time*. Scores range from 8 to 40 and are converted to percentages (e.g., score of 8 = 0%, 24 = 50%, 40 = 100%). Higher scores indicate greater social support. The MOS demonstrated good reliability in the current study (Table 2).

#### Generalized Anxiety Symptoms

The 7-item anxiety scale (GAD-7) by Spitzer et al. (2006) was used to assess symptoms of anxiety, and is also assessed at all Waves of the TTM Study. Participants responded to items assessing the frequency of various anxiety symptoms over the past two weeks on a 4-point Likert scale ranging from (0) *not at all* to (3) *nearly every day*. Higher total scores indicate greater anxiety symptoms. The GAD-7 provided good internal reliability in the current study (Table 2).

#### Depressive Symptoms

The Patient Health Questionnaire-9 (PHQ-9) by (Kroenke et al., 2001) was used to measure depressive symptoms, and is also assessed at all Waves of the TTM Study. Participants respond to items assessing the frequency of various depression symptoms over the past two weeks on a four-point Likert scale ranging from (0) *not at all* to (3) *almost everyday*. Higher total scores indicate greater depressive symptoms. The PHQ-9 provided good internal reliability in the current study (Table 2).

#### Prior Gambling Frequency/Engagement

Gambling activity was measured using a purpose-developed self-report scale regarding eight forms of gambling: EGMs, horse racing, sports, greyhound racing, casino games, poker, esports, and fantasy sports. Participants responded to each of these different types of gambling on an 8-point Likert scale ranging from (0) *never* to (7) *4 or more times a week*. Categories were collapsed to enable each category to have enough sample size. EGMs, horse racing, sports betting, and greyhound racing collapsed to a 5-point scale corresponding to: (0) *never*, (1) *once or twice a year*, (2) *a few times a year*, (3) *once to 3 times a month*, and (4) *once or more times a week*. Casino games, poker, eSports, and fantasy sports gambling were made dichotomous (i.e., never engaged in vs. engaged in) due to the infrequency of these types of gambling (i.e., < 2% engaged on a monthly or more basis). Gambling types with a range of frequencies were described as “gambling frequency”, while gambling types on a dichotomous scale were recorded as “gambling engagement”. These measures were introduced at Wave 3 and Wave 4 of the TTM Study.

### Statistical Analysis

To provide further rationale for investigating problem gambling in young men, chi-square and Fisher’s exact chi-square tests were conducted to identify differences in gambling frequency and predictor variables between young men and a sample of older men (aged > 25).

Hierarchical multiple ordinal logistic regressions (HMOLR) were conducted to investigate whether any psychosocial factors increased the likelihood for an individual to report greater problematic gambling behaviors. Hierarchical regressions allowed an investigation for whether specific factors played a greater role in explaining problematic gambling than previous steps (i.e., a significant improvement in model fit). At step 1, current demographic variables (i.e., age and socioeconomic status) were included as covariates. Step 2 included conformity to masculine norms. Step 3 included previous health-related behaviors variables (i.e., Wave 3 AUDIT scores and smoking status). Step 4 included previous social connectedness and mental health variables (i.e., Wave 3 loneliness, social support, wellbeing, anxiety, and depression). Step 5 included previous gambling frequency/engagement variables (e.g., Wave 3 frequency of sports gambling).

Last, a separate multiple ordinal logistic regression was conducted, which included the participants’ problematic gambling behavior severity from Wave 3 in addition to the predictors from Step 5. This was not part of the five-step HMOLR model, as not all participants completed the PGSI at Wave 3 (*n* = 215). However, this additional analysis allowed us to control for ‘baseline’ problematic gambling, proving a robust investigation into whether psychosocial factors could not only relate to problematic gambling but also predict changes in problematic gambling from Wave 3 to 4.

The assumption of proportional odds was met for all models (according to the Brant-Wald test). Cox-Snell's, Nagelkerke’s, and McFadden’s R^2^ were calculated for each model. For the ordinal regressions, inference testing (i.e., the identification of significant predictors) was determined using profile likelihood 95% confidence intervals (CIs) to account for asymmetric intervals inherent in ordinal logistic regression, rather than the *p* value (Christensen, 2018). However, we also report the *p* value for completeness, though this must be interpreted with caution as it does not account for this asymmetry.

### Transparency and Openness

We report how we determined our sample size, all data exclusions, and all measures in the study. The data used in this study is publicly available (upon approval by the data custodians) as part of the *Ten to Men* study (https://aifs.gov.au/research_programs/ten-men). As the *Ten to Men* study is the only longitudinal cohort study on male health, there have been other reports and outputs that have looked at patterns of gambling frequency and associated risk factors amongst the full cohort spanning 18 to 65 years (e.g., Mancini, 2025). However, no study has looked at this same set of variables, nor constrained the investigation to the specific cohort of young men aged 18 – 25 years. In other words, though data for the current study were collected as part of a larger longitudinal data collection, this work is uniquely differentiated from past work based on the sample selection, variable selection, and methodological approach. All analyses were conducted using *RStudio* version 4.4.1 (R Studio Team, 2024). Code is available on OSF (https://osf.io/djqc6/?view_only=a6e4ba2fd61c471bbaaef05e1122f9be; this link is for blinded reviewers). This study was not pre-registered in a public repository.

## Results

### Gambling Characteristics of Men Aged 18–25 years Compared to Their Older Counterparts

Chi-squares tests were performed to evaluate the assumption that the current sample of young men (≤ 25 years) differed from their older counterparts (> 25 years) on the measures of gambling frequency and psychosocial predictors. A comparison group of 1838 men from the same cohort study aged 25 years and over were included to verify potential differences in younger men. Descriptive statistics for the study variables can be found in Table 2, and descriptive statistics for gambling behaviors can be found in Table 3. Several key differences between the groups were identified, supporting our decision to isolate this subgroup for the analysis. Compared to their older counterparts, younger men reported significantly higher conformity to masculine norms, loneliness, social support, anxiety, and depression, and lower rates of wellbeing. Significant differences were also observed across problem gambling severity and frequency of gambling behaviors. A higher proportion of young men were in the moderate (3.8%) and problematic (3.4%) risk categories for the PGSI compared to older men (3.2% and 1.6%, respectively). Young men had also participated in all forms of gambling, beside horse betting, more often than older men. Correlations between study variables are presented in Table 4.

**Table 3:** Gambling behaviors of Australian men aged 18–25 years young (*n* = 265) and Australian men aged 25 years and over (*n* = 1838)

	Younger men (18–25 years)	Older men (25 years +)
Problem Gambling Severity Category (*p* = .045)		
No Risk	72.1%	78.7%
Low Risk	20.8%	16.5%
Moderate Risk	3.8%	3.2%
Problematic Risk	3.4%	1.6%
Frequency of EGMs (*p* < .001)		
Never	45.3%	59.5%
Once or twice a year	26.0%	20.9%
A few times a year	18.9%	11.0%
1–3 times a month	7.2%	5.5%
1 + a week	2.6%	3.1%
Frequency of horse betting (*p* < .001)		
Never	57.0%	43.1%
Once or twice a year	18.9%	28.6%
A few times a year	11.7%	10.0%
1–3 times a month	4.9%	7.2%
1 + a week	7.6%	11.1%
Frequency of sports betting (*p* < .001)		
Never	50.9%	63.6%
Once or twice a year	14.0%	13.8%
A few times a year	16.2%	9.3%
1–3 times a month	6.4%	7.5%
1 + a week	12.5%	5.9%
Frequency of greyhound betting (*p* = .003)		
Never	72.1%	80.5%
Once or twice a year	8.3%	7.8%
A few times a year	8.7%	5.2%
1–3 times a month	7.2%	3.4%
1 + a week	3.8%	3.1%
Ever engaged in the casino (*p* < .001)		
No	66.4%	76.9%
Yes	33.6%	23.1%
Ever engaged in poker (*p* < .001)		
No	76.6%	89.7%
Yes	23.4%	10.3%
Ever engaged in esports betting (*p* = .002)		
No	94.0%	97.6%
Yes	6.0%	2.4%
Ever engaged in fantasy better (*p* = .002)		
No	94.3%	97.8%
Yes	5.7%	2.2%

The *p*-values provided were calculated using Fisher’s exact chi-square test to examine whether gambling behaviors differed significantly between younger and older men

*EGMs* Electronic Gambling Machines

**Table 4:** Correlations. Between study variables (*N* = 265)

	1	2	3	4	5	6	7	8	9
1.PGSI	-								
2.Age	-.04	-							
3.SES	-.07	.00	-						
4.CMNI	.14*	.03	-.06	-					
5.AUDIT	.10	.07	-.01	.22**	-				
6.Loneliness	.04	-.07	.04	**.14**	**.15**	-			
7.Social support	-.02	**.12**	.08	-.07	**-.14**	-.46**	-		
8.Wellbeing	-.05	-.04	**.15**	-.06	-.23**	-.60**	.49**	-	
9.Anxiety	.06	.00	-.02	.05	.17*	.53**	-.37**	-.62**	-
10.Depression	**.11**	-.03	-.08	.08	.18*	.61**	-.43**	-.71**	.77**
11.EGMs	.17*	.01	-.09	.09	.19**	-.02	-.02	-.07	**.10**
12.Horses	.29**	.04	.00	.17**	.25**	-.03	.03	.07	-.01
13.Sports	.31**	.07	.01	.14*	.19**	**-.11**	.04	**.10**	**-.11**
14.Greyhounds	.33**	.01	.00	.13*	.18**	-.06	.06	.08	-.03
15.Casino	.12	.05	.04	.09	.27**	-.02	.01	-.06	.01
16.Poker	.14	-.03	-.02	.05	.19*	.01	-.05	.00	-.02
17.Esports	.23	-.04	-.04	.05	.02	.06	-.04	-.10	.04
18.Fantasy	.27	.08	.05	.10	.06	-.02	.09	.06	.00

	10	11	12	13	14	15	16	17
1.PGSI								
2.Age								
3.SES								
4.CMNI								
5.AUDIT								
6.Loneliness								
7.Social support								
8.Wellbeing								
9.Anxiety								
10.Depression	-							
11.EGMs	.05	-						
12.Horses	-.07	.32**	-					
13.Sports	-.13*	.23**	.58**	-				
14.Greyhounds	-.08	.31**	.74**	.56**	-			
15.Casino	-.03	.31**	.34**	.31**	.26**	-		
16.Poker	-.06	.15	.24*	.23*	.17	.34**	-	
17.Esports	.08	.00	.27	.41*	.26	.00	.11	-
18.Fantasy	-.08	.11	.49**	.45*	**.32**	.20*	.16*	**.20**

Pearson correlations were used for continuous-continuous pairs; Kendall tau correlations were used for ordinal-continuous and ordinal-ordinal pairs; point-biserial correlations were used for binary-continuous pairs; rank biserial correlations were used for binary-ordinal pairs; phi coefficients were used for binary-binary pairs

Bold indicates *p* < .05; *indicates *p* < .01; **indicates *p* < .001; PGSI = Problem Gambling Severity Index (category); *SES* Socioeconomic status (decile), *CMNI* Conformity to masculinity norms, *AUDIT* Alcohol use disorder inventory test, *Social* Social support, *EGMs* Electronic Gambling Machines

### Hierarchical Multiple Ordinal Logistic Regression (HMOLR) Predicting Problem Gambling Severity

The results of the HMOLR are reported in Table 5. The first step of the HMOLR included demographic information (i.e., age and SES). The demographic variables did not significantly predict problem gambling severity (*χ*^*2*^(2) = 2.60, *p* < 0.272; R^2^ = 0.00–0.01).

**Table 5:** Hierarchical negative binomial regression statistics for predicting problem gambling severity from relevant variables (*N* = 265)

	*OR* [95% CI for OR]	*B*	SE	*p*
*Step 1: Demographic variables* (McFadden = .01, Cox & Snell = .01, Nagelkerke = .01)				
Age	0.95 [0.84, 1.07]	−0.05	0.06	.426
Socioeconomic status	0.93 [0.85, 1.03]	−0.07	0.05	.158
*Step 2: Conformity to masculine norms* (McFadden = .02, Cox & Snell = .03, Nagelkerke = .04)				
Age	0.95 [0.85, 1.07]	−0.05	0.06	.425
Socioeconomic status	0.94 [0.85, 1.04]	−0.06	0.05	.209
Conformity to masculine norms	**1.06 [1.01, 1.12]**	**0.06**	**0.03**	**.022**
*Step 3: Health related behaviors* (McFadden = .03, Cox & Snell = .05, Nagelkerke = .06)				
Age	0.94 [0.84, 1.06]	−0.06	0.06	.339
Socioeconomic disadvantage	0.94 [0.86, 1.04]	−0.06	0.05	.231
Conformity to masculine norms	**1.06 [1.00, 1.11]**	**0.05**	**0.03**	**.038**
Problem Alcohol use	1.03 [0.97, 1.10]	0.03	0.03	.272
Smoking - Cigarettes	1.71 [0.82, 3.46]	0.54	0.37	.143
*Step 4: Social connectedness and Mental health* (McFadden = .04, Cox & Snell = .07, Nagelkerke = .08)				
Age	0.95 [0.84, 1.07]	−0.05	0.06	.387
Socioeconomic disadvantage	0.95 [0.86, 1.05]	−0.05	0.05	.335
Conformity to masculine norms	**1.06 [1.00, 1.12]**	**0.06**	**0.03**	**.037**
Problem Alcohol use	1.03 [0.97, 1.09]	0.03	0.03	.346
Smoking - Cigarettes	1.73 [0.82, 3.58]	0.05	0.38	.144
Loneliness	0.98 [0.90, 1.08]	−0.02	0.05	.751
Social support	1.01 [0.97, 1.05]	0.01	0.02	.640
Subjective wellbeing	1.00 [0.97, 1.03]	0.00	0.01	.990
Generalized anxiety symptoms	0.92 [0.83, 1.00]	−0.09	0.05	.067
Depressive symptoms	**1.10 [1.01, 1.20]**	**0.10**	**0.04**	**.023**
*Step 5: Gambling Frequency* (McFadden = .17, Cox & Snell = .24, Nagelkerke = .30)				
Age	0.95 [0.84, 1.09]	−0.05	0.07	.452
Socioeconomic disadvantage	0.95 [0.85, 1.06]	−0.05	0.06	.341
Conformity to masculine norms	1.04 [0.98, 1.11]	0.04	0.03	.210
Problem Alcohol use	0.97 [0.90, 1.04]	−0.04	0.04	.338
Smoking - Cigarettes	1.66 [0.73, 3.69]	0.51	0.41	.216
Loneliness	1.00 [0.91, 1.11]	0.00	0.05	.944
Social support	1.01 [0.97, 1.06]	0.01	0.02	.610
Subjective wellbeing	1.00 [0.97, 1.03]	0.00	0.02	.820
Generalized anxiety symptoms	**0.89 [0.79, 0.99]**	**−0.12**	**0.06**	**.032**
Depressive symptoms	**1.18 [1.07, 1.30]**	**0.16**	**0.05**	**.001**
EGMs frequency	1.31 [0.96, 1.78]	0.27	0.16	.081
Horse racing gambling frequency	1.01 [0.64, 1.59]	0.01	0.23	.973
Sports gambling frequency	**1.64 [1.22, 2.20]**	**0.49**	**0.15**	** < .001**
Greyhound gambling frequency	1.27 [0.81, 1.99]	0.24	0.23	.298
Casino engagement	0.73 [0.35, 1.47]	−0.31	0.36	.387
Poker engagement	1.81 [0.87, 3.74]	0.59	0.37	.110
Esports gambling engagement	0.86 [0.25, 2.66]	−0.15	0.60	.797
Fantasy sports gambling engagement	2.34 [0.67, 7.58]	0.85	0.02	.820

Significant predictors, as indicated by 95% CIs that do not contain the null value of 1, are presented in bold

In Step 2, conformity to masculine norms was introduced as an additional predictor of problem gambling severity. The inclusion of this predictor significantly improved from the previous model (Δ*χ*^*2*^(1) = 5.41, *p* = 0.020) and explained an additional 1%—3% of problem gambling risk (ΔR^2^ = 0.01–0.03). The demographic variables did not influence problematic gambling, although, a one unit increase in conformity to masculine norms increased the odds of future problematic gambling by 6% (OR = 1.06, 95% CI = [1.01, 1.12]).

In Step 3, health-related behaviors (i.e., audit scores and whether currently smoking) were introduced as additional predictor variables. The inclusion of these variables did not provide a significant improvement to the model’s ability in predict future problematic gambling (Δ*χ*^*2*^(2) = 4.28, *p* = 0.117 ΔR^2^ = 0.01–0.02). Conformity to masculine norms continued to be a significant predictor of problem gambling severity (OR = 1.06, 95% CI = [1.00, 1.11]).

In Step 4, social connectedness and mental health variables were introduced. These variables did not provide a significant improvement to the previous model (Δ*χ*^*2*^(5) = 6.26, *p* = 0.282, ΔR^2^ = 0.01–0.03). Conformity to masculine norms remained a statistically significant predictor of problem gambling severity (OR = 1.06, 95% CI = [1.00, 1.12]). While the Step 4 model did not show significant improvements, a one-unit increase in depression scores increased the odds of problematic gambling one year later by 10% (OR = 1.10; 95% CI = [1.01, 1.20]).

**Fig. 1:**
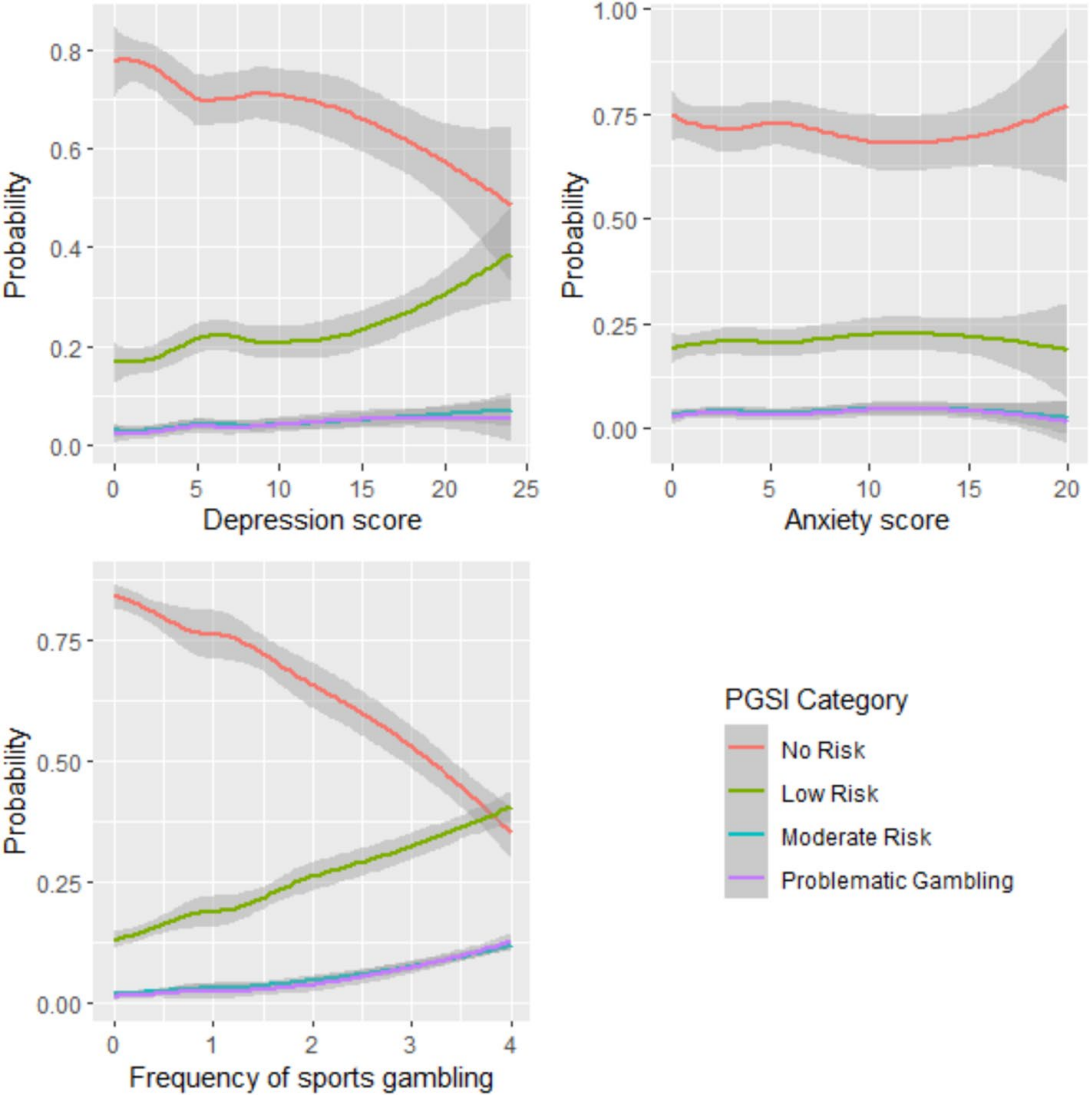
The probability of an individual belonging to different risks of problematic gambling groups in Wave 4 based on their depression score, anxiety score, and frequency of sports gambling at Wave 3. *Note:* for frequency of sport gambling: 0 = Never used; 1 = once a year; 2 = a few times a year; 3 = 1–3 times a month; 4 = weekly or more

In Step 5, gambling frequency/engagement (i.e., how often or whether young men engage in different gambling types, such as sports gambling) were introduced. The inclusion of these gambling frequencies provided a significant improvement to the previous model (Δ*χ*^*2*^(8) = 54.19, *p* < 0.001) and collectively explained an additional 13%—22% of problem gambling risk (ΔR^2^ = 0.13–0.22). Controlling for the effects of previous gambling frequency, the effect of masculine norms was no longer significant (OR = 1.04, 95% CI = [0.98, 1.11]). However, depression continued to increase the odds of later problem gambling (OR = 1.18 95% CI = [1.07, 1.30]). Additionally, individuals with a one-unit greater anxiety score had 11% less odds of later problem gambling (OR = 0.89, 95% CI = [0.79, 0.99]). When inspecting specific gambling forms, only the frequency of sports gambling was uniquely related to an increased risk of problematic gambling. Young men who showed a one-unit increase (e.g., a few times a year compared to 1–3 times a month) had a 64% increased odds of later problematic gambling (OR = 1.63, 95% CI = [1.22, 2.20]). Figure 1 provides the probabilities of problematic gambling risk across the range of depression and anxiety scores and sport gambling frequency.

### Accounting for Prior Problem Gambling Severity

Last, to examine whether these effects predicted changes in problem gambling, participants’ PGSI scores at Wave 3 was introduced as a predictor. As not all participants completed the PGSI at Wave 3, this analysis had a smaller sample size (*n* = 215) which fell short of our recommended sample size based on the power analysis (*N* = 244) and is a separate analysis from the previous hierarchical regression.^1^

**Fig. 2:**
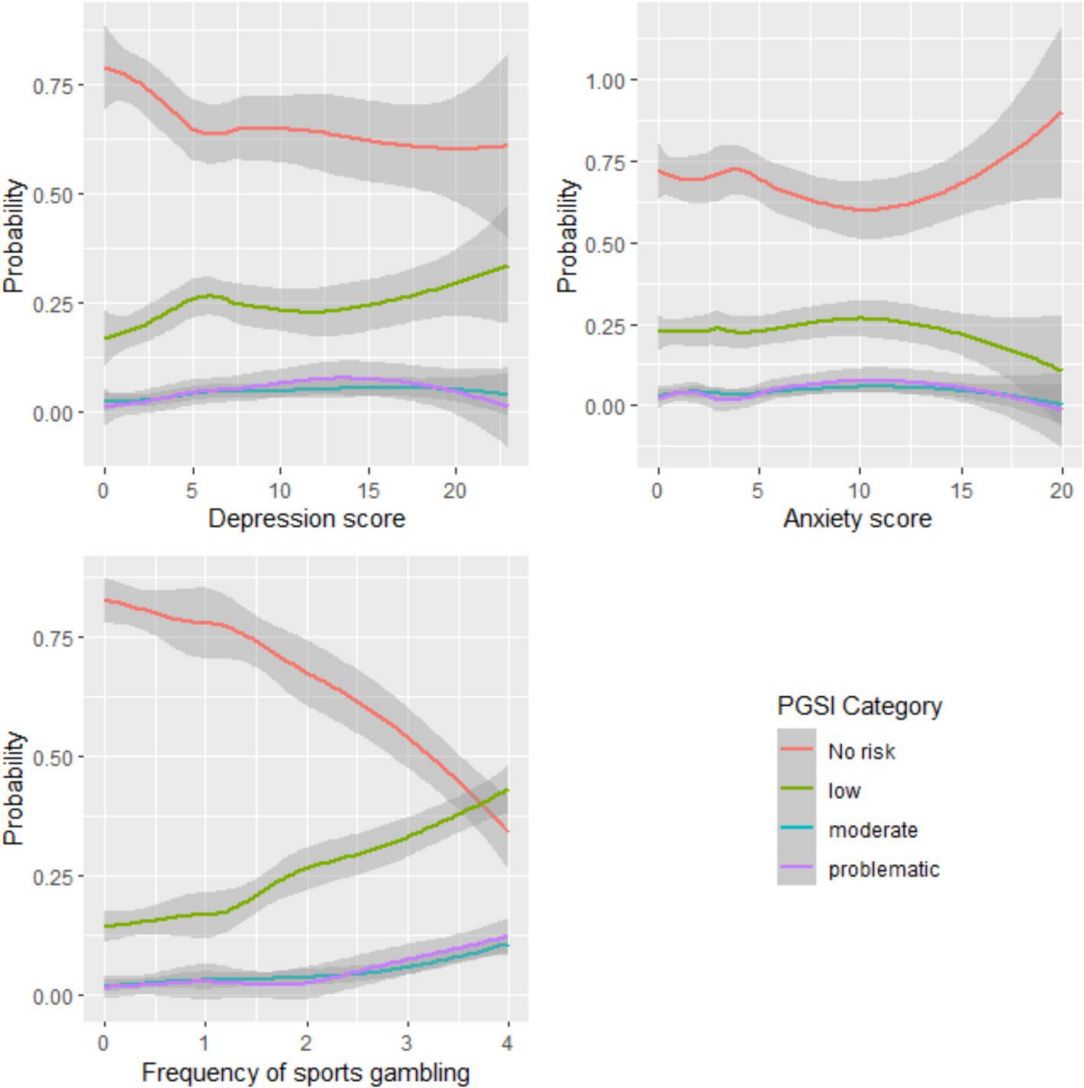
The probability of an individual belonging to different risks of problematic gambling groups in Wave 4 based on their depression score, anxiety score, and frequency of sports gambling, controlling for problematic gambling severity at Wave 3. *Note:* for frequency of sport gambling: 0 = Never used; 1 = once a year; 2 = a few times a year; 3 = 1–3 times a month; 4 = weekly or more

The results were broadly consistent with those observed in the HMOLR (see Table 6 and Fig. 2). Higher levels of anxiety predicted lower odds of increasing problem gambling severity between Wave 3 and 4 (OR = 0.88, 95% CI = 0.769, 0.997]). Consistent with the HMOLR, greater depression (OR = 1.19, 95% CI = [1.06, 1.35]), and sports gambling (OR = 1.45, 95% CI = [1.04, 2.04]) at Wave 3 had greater odds of increasing their problem gambling severity by Wave 4. Interestingly, fantasy sports gambling was also a significant predictor in this model, controlling for Wave 3 problem gambling severity (OR = 3.97, 95% CI = 1.01, 14.48]).

**Table 6:** Negative binomial regression statistics for predicting problem gambling severity from relevant variables after accounting for prior problem gambling severity (*N* = 215)

Variable	*OR* [95% CI for OR]	*B*	SE	*p*
Age	0.94 [0.80, 1.09]	−0.06	0.08	.414
Socioeconomic disadvantage	1.00 [0.88, 1.14]	0.00	0.07	.958
Conformity to masculine norms	1.00 [0.93, 1.08]	0.00	0.04	.978
Problem Alcohol use	1.03 [0.95, 1.12]	0.03	0.04	.483
Smoking - Cigarettes	2.16 [0.88, 5.25]	0.77	0.45	.089
Loneliness	0.93 [0.82, 1.04]	−0.08	0.06	.212
Social support	1.00 [0.95, 1.05]	0.00	0.03	.901
Subjective wellbeing	1.00 [0.97, 1.04]	0.00	0.02	.878
**Generalized anxiety symptoms**	0.88 [0.77, 1.00]*	**−0.13**	**0.07**	**.052**
**Depressive symptoms**	**1.19 [1.06, 1.35]**	**0.18**	**0.06**	**.003**
EGMs frequency	1.25 [0.90, 1.75]	0.23	0.17	.184
Horse racing gambling frequency	1.11 [0.69, 1.78]	0.10	0.24	.669
**Sports gambling frequency**	**1.45 [1.04, 2.04]**	**0.37**	**0.17**	**.028**
Greyhound gambling frequency	0.84 [0.52, 1.35]	−0.17	0.24	.479
Casino engagement	0.73 [0.33, 1.57]	−0.31	0.39	.427
Poker engagement	1.66 [0.75, 3.65]	0.51	0.40	.205
Esports gambling engagement	0.42 [0.11, 1.43]	−0.88	0.64	.176
**Fantasy sports gambling engagement**	**3.97 [1.01, 14.48]**	**1.38**	**0.67**	**.040**
**Wave 3 Problem gambling linear effect**	**57.15 [13.91, 258.04]**	**4.05**	**0.74**	** < .001**
**Wave 3 Problem gambling quadratic effect**	**0.29 [0.08, 0.95]**	**−1.25**	**0.61**	**.040**
**Wave 3 Problem gambling cubic effect**	**0.23 [0.06, 0.80]**	**−1.48**	**0.66**	**.024**

Significant predictors, as indicated by 95% CIs that do not contain the null value of 1, are presented in bold. *Generalized anxiety symptoms marked as significant as the 95% CI does not contain the null value [0.769, 0.997]

## Discussion

The aim of this study was to identify the psychosocial, health-related, and behavioral predictors of problem gambling symptoms in young Australian men aged 18–25 years. Study data was drawn from eligible participants in the *Ten to Men* study (Currier et al., 2016), which remains the largest Australian longitudinal survey on male health and wellbeing. Congruent with Hypothesis One, we found that the combination of variables included in the final model (demographics, masculine norm conformity, health-related behaviors, social connectedness and mental health, and prior gambling frequency) explained a moderate-to-large (17–30%) degree of variance in problem gambling severity. Contrary to Hypothesis Two, only four of the 18 predictors included in the HMOLR model were found to explain unique variance in problem gambling severity, once Wave 3 gambling severity was accounted for. Specifically, higher levels of depression, lower levels of anxiety, and a higher frequency of engagement in *sports* and *fantasy sports* gambling predicted higher rates of problem gambling severity. This pattern of findings differs from previously published gambling research using the full *TTM* cohort (Mancini, 2025), indicating potentially age-dependent effects and emphasizing the need for further investigation into the gambling-related experiences of young men. Our focus on young men is also driven by the growing evidence that has found young men to be at disproportionately greater risk for gambling-related harms relative to older men or women in the same age range (Benakovic et al., 2024). The key findings and their implications from the current study are interpreted below.

### *Sports *and *Fantasy Sports* Gambling Uniquely Predict Problem Gambling in Young Men

Our analysis found that betting on organized sports (i.e., *sports gambling*) within the 12-months prior to Wave 3 data intake was the strongest predictor of problem gambling severity and appeared to account for the most unique variance in this outcome (at Wave 4). To our knowledge, this study is the first to differentiate *sports* gambling from *fantasy sports* gambling, identifying both as unique risk factors for problem gambling severity among young Australian men. However, it is important to acknowledge that fantasy sports are inextricably linked from organized sports. As fantasy sports routinely award ‘points’ for players and teams based on their performance in real life, isolating fantasy sports from organized sports is not possible. However, the results also speak to the potential risk that *fantasy sports* impose as a potential ‘gateway’ to sports gambling (Houghton et al., 2019; Martin et al., 2016; Pickering et al., 2016). The popularity of fantasy sports as a conceivably ‘safe’ way for men to derive greater excitement or social connection (by joining a league with friends) from viewing organized sports should be tempered against the potential risk that this poses to the onset of problem gambling, as identified in the current study and in prior research (Martin & Nelson, 2014; Pickering et al., 2016).

These results add context to recently published work by Mancini (2025) who, using an aggregated measure of ‘overall’ gambling frequency, predicted future problem gambling severity in a larger subset of the *Ten to Men* cohort (*N* = 2,018, mean age ~ 44 years). However, those findings did not examine the differential effects of *type* of gambling activity, nor whether these varied as a function of age. The current findings addressed this gap. Younger males reported a different pattern of gambling activity to their older counterparts, and sports gambling was the only form of gambling predictive of problem gambling severity in the final model.

Unexpectedly, none of the other forms of gambling behaviors that were measured (i.e., EGMs, horse racing, greyhound racing, casino, poker, and esports) could account for any further variance in problem gambling severity after accounting for the effect of sports gambling. This finding is discrepant with past Australian literature, where EGMs were shown to be the behavior most strongly accounting for gambling problems in a large population-based study (Browne et al., 2023). One compelling explanation for this discrepancy is that our current study focused exclusively on the gambling-related experiences of young men, where this earlier research had not necessarily stratified results by age and/or gender. Young men may engage in sports (including fantasy sports) betting more frequently than older adults (Armstrong & Carroll, 2017; Australian Gambling Research Centre, 2023), although they may not necessarily engage less than older men in other forms of gambling (Table 3). Accordingly, the reliance on such aggregated findings may inadvertently diminish the effectiveness of gambling-related harm-reduction initiatives designed to penetrate vulnerable groups, including young men. For example, attempting to reduce the gambling-related harms from EGMs may yield limited benefits for younger men, considering that our results suggest that they are more likely to experience higher rates of problem gambling following higher rates of participation in sports gambling. These results help justify the development of tailored initiatives that target the unique gambling-related behaviors of young men, with sports gambling participation identified as a factor salient to gambling problems.

Our results are nested in a larger body of interdisciplinary research that has identified sports gambling as a significant public health concern that disproportionately impacts younger men. Prior research has identified that males are more likely to gamble on ‘skill-based’ activities, relative to young females (Boldero & Bell, 2012). Additionally, younger adults more often engage in gambling behaviors that have a more ‘skill-based’ component compared to older adults who prefer ‘chance-based’ gambling activities (Stevens & Young, 2010). This intersection of age and gender means that younger men may find sports gambling a particularly enticing form of gambling and, by extension, are vulnerable to its consequences (Benakovic et al., 2024).

The predisposition that young men have for skill-based forms of gambling may be further amplified when organized sports are used as the medium for gambling activity. This may also partly explain why sports gambling (including fantasy sports), but no other form of gambling activity, were predictive of gambling problems in our young male sample. Though changing, organized sport persists as an industry that is heavily male-dominated, both with respect to its athletes and enthusiasts (Toffoletti & Palmer, 2019), which has heavily integrated betting through sponsorship and advertisements (Australian Gambling Research, C, 2023). The promotion of sports betting into organized sport through sponsorship and advertisements in Australia has been identified as an issue by the community and regulatory agencies, which has resulted in several campaigns to address this. A noteworthy example includes the New South Wales State Government’s *Reclaim the Game* campaign (New South Wales State Government, 2024).

Gambling companies have been known to leverage young men’s interests in organized sports to incite gambling participation. Noteworthy examples include sports gambling marketing campaigns designed to appeal to a younger male demographic, an abundance of sports betting advertisements during televized sports (and now through social media and targeted marketing), sponsorship of sporting teams and venues by gambling agencies, and the use of known sporting personalities (e.g., current and former athletes) to endorse sports gambling platforms (Gainsbury et al., 2015, 2016). Tussey (2022) recently described the evolution of sports betting into a normalized part of organized sports viewership, where “gambling on sports is a transaction that allows fans to play along with other sports fans” (p.364). This aligns with work that describes how sports betting is now become a standard part of young men’s sport fan rituals (Dietrich & Raj, 2023), which may also increase risk for psychosocial harm.

More traditional forms of marketing are now often supplemented by technological innovations that provide gambling operators with innovative ways to target specific individuals based on demographic (e.g., young men) or behavioral (e.g., engages in sports and/or gambling-related content online) factors known to increase gambling risk (Rothwell, 2023). Additionally, the rising popularity of ‘tipsters’ – individuals who gain online following for providing betting advice or showcasing their gambling behaviors – might also help to normalize sports gambling (Gonzálvez-Vallés et al., 2021). This may leave certain impressionable groups (e.g., young people) susceptible to these behaviors. These problem behaviors may be further maintained if the harm reduction or prevention campaigns are delivered in a modality that do not reach the intended target audience. For example, campaigns shown on free-to-air television may not reach many young men who are using streaming services or social media to consume sport and/or gambling-related content. Accordingly, the identification of *how* men access various gambling-related stimuli may be essential for the development of effective campaigns.

By creating the perception that sports gambling is a normalized behavior may further increase one’s likelihood to engage in this behavior, subsequently increasing their risk for future gambling problems (Martin et al., 2010). Our results add to earlier literature outlining the potential harms of sports gambling behavior for young men, revealing that this was the only form of gambling activity that could predict problem gambling severity in our sample. Future research that aims to better isolate young men’s experiences of sports gambling (e.g., its integration with organized sport, links to masculine behavior, the ubiquity of sports gambling advertising) relative to other forms of gambling can help to develop effective methods to mitigate this risk.

### Low Anxiety and High Depression Further Predict Problem Gambling in Young Men

The distinction between anxiety and depression as risk factors for problem gambling in the current study warrants closer interrogation. These internalizing symptoms remained important predictors of problem gambling severity, even after accounting for prior problem gambling severity. Despite the strong and positive correlation between anxious and depressive symptoms (*r* = 0.77), we found that they were *inversely* predictive of problem gambling severity in the final step of the HMOLR, and the additional analysis controlling for prior problem gambling severity. Specifically, *low* anxiety and *high* depressive symptoms were associated with higher levels of problem gambling severity. These inverse findings may reflect suppression effects, whereby these effects occur once accounting for the shared variance between anxiety and depression (i.e., general psychological distress), warranting further interrogation.

Conceptually, the processes that link symptoms of anxiety and depression with problem gambling may be distinct. People who are highly anxious may be more averse to risk-taking behaviors like gambling, which aligns with our current pattern of results. Alternatively, the experience of depression may be characterized by low mood and no longer gaining enjoyment or pleasure from previously enjoyable activities. To provide an example within the current context, it may be hypothesized that depressed young men who no longer enjoy watching organized sport might turn toward sports gambling to make them more enjoyable. This is also congruent with prior evidence in the clinical literature that describes problem gambling behaviors as the maladaptive attempt by depressed individuals to upregulate their low mood (Vaughan & Flack, 2022). This may also serve to maintain, if not worsen, their depressed state in the likely event that these individuals continue to fall further into problem gambling. Future studies that include multiple (ideally three or more) timepoints would be able to further examine the interplay between anxiety and depression with gambling behaviors and problems over time – including analyses that can more robustly establish causal inference.

Our current findings were derived from a community sample of young Australian men. Accordingly, we enlisted a dimensional approach to the assessment of anxious and depressive symptomology. This also means that there was an underrepresentation of young men experiencing clinically significant anxiety and/or depression, though we acknowledge that these individuals are likely to be overrepresented amongst a cohort of young men experiencing gambling problems. This highlights the need for future research that considers the expression of anxious and depressive disorders in younger males, and how this intersection between psychopathology, age, and gender, may trigger and maintain gambling problems. The current results may be complementary to these future efforts, as the experience of depression (and their consequences) are not only reserved for clinical subgroups. Rather, the identification of anxious and depressive symptoms as unique predictors of problem gambling among a community sample of young men highlights the potential value of targeting mental health as part of wider efforts to prevent gambling-related harm in this group.

### Implications for Policy and Practice

In Australia, policymakers are tasked with the responsibility of regulating the gambling industry and developing effective responses to mitigate gambling-related harms. Recent examples of legislative changes include banning the promotion of ‘live’ betting odds and bonuses offered to new customers (Dietrich & Raj, 2023). Some restrictions around the timing and content of marketing strategies have also been implemented, which aligns with prior work that has identified that young people are more likely to gamble problematically after seeing a high volume of sports gambling advertisements (Freund et al., 2022). Using some of the most recently available longitudinal data concerning the health and wellbeing of Australian males, we found that sports gambling behavior persists as a unique and significant determinant of future gambling problems. This suggests that ongoing policy-level innovation may be required if there is a motivation to prevent gambling-related harm among younger males. The relevance of psychopathology as predictors of problem gambling beyond past gambling frequency highlights the need for initiatives that consider how to mitigate psychosocial risk (Benakovic et al., 2024).

The identification of depressive symptoms as a risk factor for gambling problems also suggests that efforts to reduce these symptoms may be a critical component of gambling harm-reduction initiatives. For example, psychoeducation on the role of sensation-seeking may help younger men to understand how the symptoms of depression may increase the risk for gambling problems. Additionally, the promotion of alternative and adaptive strategies that can alleviate low mood among younger men may prove effective in reducing the risk of turning to gambling as a means to upregulate negative affect (Atkinson et al., 2012). This aligns with recent evidence that suggests that psychological interventions to treat problem gambling can also be delivered online, highlighting the potential scalability of digital approaches that might be able to meet growing demand (Augner et al., 2022).

The potential to optimize these interventions for younger men may also be explored through the adoption of ‘masculinities’ models of mental health that can help increase the uptake and effectiveness of treatment for men seeking support (Seidler et al., 2017, 2024a, b). These efforts should not be considered as reinforcing unhelpful gender stereotypes but instead, an attempt by the healthcare profession to engage with a group that has, historically, been less receptive to professional support yet, paradoxically, is in high need of this support. For men, particularly those who are resistant to conventional models of professional support, creating opportunities that use male-oriented discourse, build genuine rapport, connection and collaboration, and hold a clear goal-oriented focus have been shown to increase receptiveness to professional intervention (Rice et al., 2018; Seidler et al., 2018a, b). Additionally, these intervention efforts also need to be responsive to the dynamic and rapidly evolving nature of the gambling behaviors among young men, such as what they are gambling on, where they are gambling (e.g., new technologies and platforms), and the driving factors that encourage gambling participation (e.g., social media, paid influencer marketing campaigns).

Last, the application of a commercial determinants of health framework (Gilmore et al., 2023) may help to continue to drive policies and legislation that acknowledge the role of the gambling industry in shaping the gambling behaviors of young men through both marketing and gambling technology. Opportunities for the industry to proactively work in partnership with regulatory bodies to mitigate gambling-related harms is an evolving process; the effectiveness of which can be guided by quality evidence.

### Limitations and Directions for Future Research

There remains a paucity of longitudinal data measuring the psychosocial wellbeing and health-related behaviors of young Australian men. The *Ten to Men* study provided critical data to address this gap, though delaying the introduction of gambling-related variables (frequency and problem gambling symptoms) into Wave 3 and Wave 4 of the study meant that our final sample (*N* = 265 for the HMOLR; *n* = 215 after controlling for prior problem gambling severity) of young men aged 18 – 25 years represented only a minority of this larger cohort and may warrant some considerations about the generalizability of findings. However, while the present results highlight changes from one point of time to another, future research can further strengthen the current findings by investigating these relationships with intensive longitudinal data methodology (e.g., experience sampling methods).The current results add to an emerging body of literature which identifies the psychosocial risk factors and behavioral risk factors for problem gambling in Australian men. This may help guide the development of strategies and studies that can more deeply interrogate men’s experiences with gambling, and the factors that increase (or prevent) vulnerability to gambling-related harm.

Whilst the use of self-report measures is commonplace in large cohort studies (as well as social sciences research), it is also important to note that they may also be subject to certain biases. This is particularly relevant in gambling research, as recall biases concerning the frequency and impact of gambling activity may depart substantially from objective experiences (Heirene et al., 2022). Future studies that have access to objective data (e.g., metadata obtained from betting platforms, bank transactions) would offer more precise measures.

## Conclusions

Using data from the largest national survey of Australian male health, the current results revealed that men aged 18 – 25 years gambled more frequently than their older peers. Patterns of gambling behavior were also different. Expanding on recently published work that identified the psychosocial, health-related, and behavioral risk factors for gambling problems by Mancini (2025), our results revealed three key risk factors that could predict young men’s problem gambling severity. First, a higher frequency of engagement in *sports* and *fantasy sports* gambling were the only types of gambling activity that could account for unique variance in problem gambling severity. Second, lower rates of anxiety were associated with increased future gambling severity. Third, higher rates of depression were associated with increased gambling severity. These results suggest that young men’s gambling-related experiences are unique. Through the identification of risk and protective factors associated with problem gambling severity, our results may help to direct policy and intervention initiatives that meet the needs of young men.

## Supplementary Information

Below is the link to the electronic supplementary material.

Supplementary file1 (DOCX 59 KB)

## Footnotes

1 These results indicated that problem gambling at Wave 3 was a strong and nonlinear predictor of problem gambling at Wave 4. A substantial positive linear effect (OR = 57.15, 95% CI = [13.91, 258.04]) indicated that young men with more severe problem gambling had 57 times more odds of being in a higher problem gambling severity at Wave 4. Quadratic (OR = 0.29, 95% CI = [0.08, 0.95]) and cubic (OR = 0.23, 95% CI = 0.06, 0.80]) effects indicated that the relationship varied across the problem gambling severity, with the effect flattening as problem gambling increased (i.e., less than 57 times greater odds).

## Declarations

### Use of Existing Data

Data for this study were obtained from the *Ten to Men Study*, a publicly available longitudinal cohort study of Australian male health (https://aifs.gov.au/tentomen). However, the combination of ideas and data appearing in this manuscript are unique to this study and have not been reported elsewhere.
