# Supplementary material for: Predicting Problem Gambling in Young Men: The Impact of Sports Gambling Frequency and Internalizing Symptoms
Source: J Gambl Stud. 2025 Jun 11;41(3):1119–44. doi: 10.1007/s10899-025-10403-0 (PMC12361320; doi:10.1007/s10899-025-10403-0)
Supplement: Supplementary file 1 — Supplementary file1 (DOCX 59 KB) [file 10899_2025_10403_MOESM1_ESM.docx]

**Supplementary Material A**

Flow chart demonstrating sample selection from the full *Ten to Men* Cohort.

Total Sample at Wave 4 of the *TTM* Study when study outcome (PGSI) was administered (*N* = 7,050)

Total sample with valid outcome (PGSI Wave 4) data (*N* = 2,584)

Unable to determine value / Not applicable for Wave 4 PGSI (*N* = 4,466)

Did not provide data for:

SEIFA (*N* = 95)

CMNI (*N* = 105)

AUDIT (*N* = 175)

Smoking (*N* = 3)

Well-being (*N* = 25)

GAD (*N* = 1)

PHQ-9 (*N* = 42)

UCLA Loneliness (*N* = 26)

MOS – Social support (*N* = 8)

Gambling Frequency (*N* = 1)

Young men (i.e., < 25 years) available for the study (*N* = 265)

Total older men sample (i.e., 25 years+) for comparison analyses (*N* = 1,838)

Total sample with valid data for outcome (PGSI Wave 4) and predictor variables (*N* = 2,103)
